# Immune Tolerance Induction against Experimental Autoimmune Encephalomyelitis (EAE) Using A New PLP-B7AP Conjugate that Simultaneously Targets B7/CD28 Costimulatory Signal and TCR/MHC-II Signal

**DOI:** 10.4172/2376-0389.1000131

**Published:** 2014-12-11

**Authors:** Ahmed H Badawi, Paul Kiptoo, Teruna J Siahaan

**Affiliations:** 1 Department of Pharmaceutical Chemistry, The University of Kansas, Lawrence, KS 66047, USA; 2 KU Medical Center, The University of Kansas, Kansas City, KS 66160, USA

**Keywords:** T cell Receptor, Inflammation, Multiple Sclerosis, Bifunctional peptide inhibitor, Cytokines

## Abstract

Most of the current therapies used in the treatment of multiple sclerosis (MS) are either ineffective or have adverse side effects. As such, there is a need to develop better therapies that specifically target myelin-specific aberrant immune cells involved in CNS inflammation without compromising the general immune system. In the present study, we developed a new bifunctional peptide inhibitor (BPI) that is effective and specific. Our BPI (PLP-B7AP) is composed of an antigenic peptide from myelin proteolipid protein (PLP_139–151_) and a B7 antisense peptide (B7AP) derived from CD28 receptor. The main hypothesis is that PLP-B7AP simultaneously targets MHC-II and B7-costimulatory molecules on the surface of antigen presenting cells (APC) and possibly alters the differentiation of naïve T cells from inflammatory to regulatory phenotypes. Results showed that PLP-B7AP was very effective in suppressing experimental autoimmune encephalomyelitis (EAE) compared to various controls in a mouse model. PLP-B7AP was effective when administered both before and after disease induction. Secreted cytokines from splenocytes isolated during periods of high disease severity and remission indicated that PLP-B7AP treatment induced an increased production of anti-inflammatory cytokines and inhibited the production of pro-inflammatory cytokines. Further, analysis of cortical brain tissue sections showed that PLP-B7AP treated mice had significantly lower demyelination compared to the control group. All these taken together indicate that the T cell receptor (TCR) and the CD28 receptor can be targeted simultaneously to improve efficacy and specificity of potential MS therapeutics.

## Introduction

Inflammation of the central nervous system (CNS) is one of the main hallmarks of multiple sclerosis (MS), a neurodegenerative disease that leads to damage of neuronal axons [1]. In the case of MS, a specific immune trigger in the periphery is thought to cause antigens related to myelin to be processed by dendritic cells in the lymphoid system and presented to T-cells on the surface of antigen-presenting cells (APC); leading to activation and clonal expansion of antigen-specific T cells. These T cells can then cross the blood-brain barrier (BBB) and enter the CNS, where they are reactivated by the target antigen on myelin. Production of inflammatory cytokines causes inflammation of the myelin and subsequent extensive recruitment of phagocytic blood-borne monocytes and/or macrophages result into the formation of sclerotic plaques, a characteristic of MS. Destruction of myelin exposes the underlying axons and eventual axonal damage causes neurological disability. Depending on localization of the inflammation, MS patients experience a variety of symptoms such as sensory loss, visual impairment, muscle weakness, ataxia, and impaired balance. Experimental autoimmune encephalomyelitis (EAE) animal models have some of the disease characteristics of MS, including CNS inflammation, lesion formation, BBB breakdown, and demyelination [2]. Thus, EAE is widely used model to study novel therapies for MS. For both MS and EAE, it is believed that the inflammatory response is primarily due to the activation of CD4+ T cells. Activation of inflammatory CD4+ T cell response requires two signals that must be delivered to the T cell by APC; an antigen-specific signal and a costimulatory signal [3–5].

The antigen-specific signal, known as Signal-1, is delivered via the interaction of the T-cell receptor (TCR) on the surface of a T cell and an antigen-loaded major histocompatibility complex class-II (MHC-II) molecule on the surface of an APC [5]. The costimulatory signal, also known as Signal-2, is produced by the interaction of various molecules on the surface of T cells and APC [5–8]. The most important costimulatory signal is generated by the interaction between CD28 on CD4+ T cells and its coreceptor B7 on the surface of APC [7,8]. CD28 binds to B7 via a conserved extracellular region characterized by the MYPPPY motif [9–11]. This region involving B7/CD28 interaction has been explored in the design of targeted therapies. A bifunctional fusion protein (BFP) designed as a conjugate between the extracellular portion of B7-1 and an anti-carcinoembryonic antigen (CEA) to form B7-αCEA diabody has been shown to stimulate tumor-specific T cells [12]. B7-αCEA targets the TCR pathway by stimulating activation and proliferation of tumor-reactive T cells; thereby causing an increase in death of tumor cells [12]. CD28/B7 interaction has also been shown to be crucial for the progression of MS and EAE. Another molecule that interacts with B7 is CTLA-4, but, unlike CD28, CTLA-4 is important for inhibiting the stimulation of T cells [13]. This interaction has also become useful for designing therapies that can mimic the inhibitory signal for the suppression of inflammatory responses. Other well-studied costimulatory signals include the CD40/CD40L interaction [14] and a group of adhesion molecule interactions between T cells andAPC. Adhesion molecules are believed to strengthen the connection between both cells and, therefore, enhance the delivery of signals from the APC to the T cells. The most important pair of adhesion molecules is the intercellular adhesion molecule-1 (ICAM-1) on the APC and leukocyte function-associated antigen-1 (LFA-1) on the T cell [15].

Several therapies have been developed in an attempt to allow delivery of only an antigen-specific signal (Signal-1) in the absence of costimulatory signals (Signal-2). This has been achieved either by blocking Signal-2 [16,17] or using fixed APC primed with antigen [18,19]. Blocking Signal-2 or the absence of Signal-2 can induce anergy in T cells and lead to long-term tolerance toward a specific antigen [19–21]. Using antisense technology, Chen et al. discovered the B7AP peptide with the conserved MYPPPY sequence from the CD28 protein [22]. B7AP binds to B7 and specifically blocks the B7-CD28 interaction without affecting the B7-CTLA-4 interaction. This peptide has been used to prevent allograft rejection in mice. A larger peptide (EL-CD28) containing the conserved MYPPPY sequence from CD28 was also shown to reduce the severity of EAE [23]. Unfortunately, solely blocking B7-CD28 interactions may cause general immunosuppression because there is no antigenic selectivity of the peptide. Therefore, there is a need to alter the differentiation and proliferation of immune cells in an antigenic-specific manner for controlling immune response in autoimmune diseases.

Previously, we developed a variety of bifunctional peptide inhibitors (BPIs) that were formed by conjugating an antigenic peptide (e.g., PLP, MOG, and MBP) to peptides that inhibit interaction of LFA-1/ ICAM-1, another molecular pair responsible for initiation of the co-stimulatory signal of the immunological synapse. Extensive studies were conducted to rigorously evaluate the efficacy of these molecules [24–29]. We discovered that substituting the antigenic peptide with a non-immunogenic peptide, a scrambled peptide, or even an antigenic peptide different from the one used to induce the disease abolished EAE suppressive activity of these molecules [24,29]. The same applies to the other portion of the BPI. Replacing LABL or cIBR with a peptide that does not affect the molecular pairs (e.g., LFA-1/ICAM-1, CD28/B7, CTLA-4/B7 etc.) responsible for the co-stimulatory signal tremendously decreased the pharmacological activities of these molecules.

**Figure 1:**
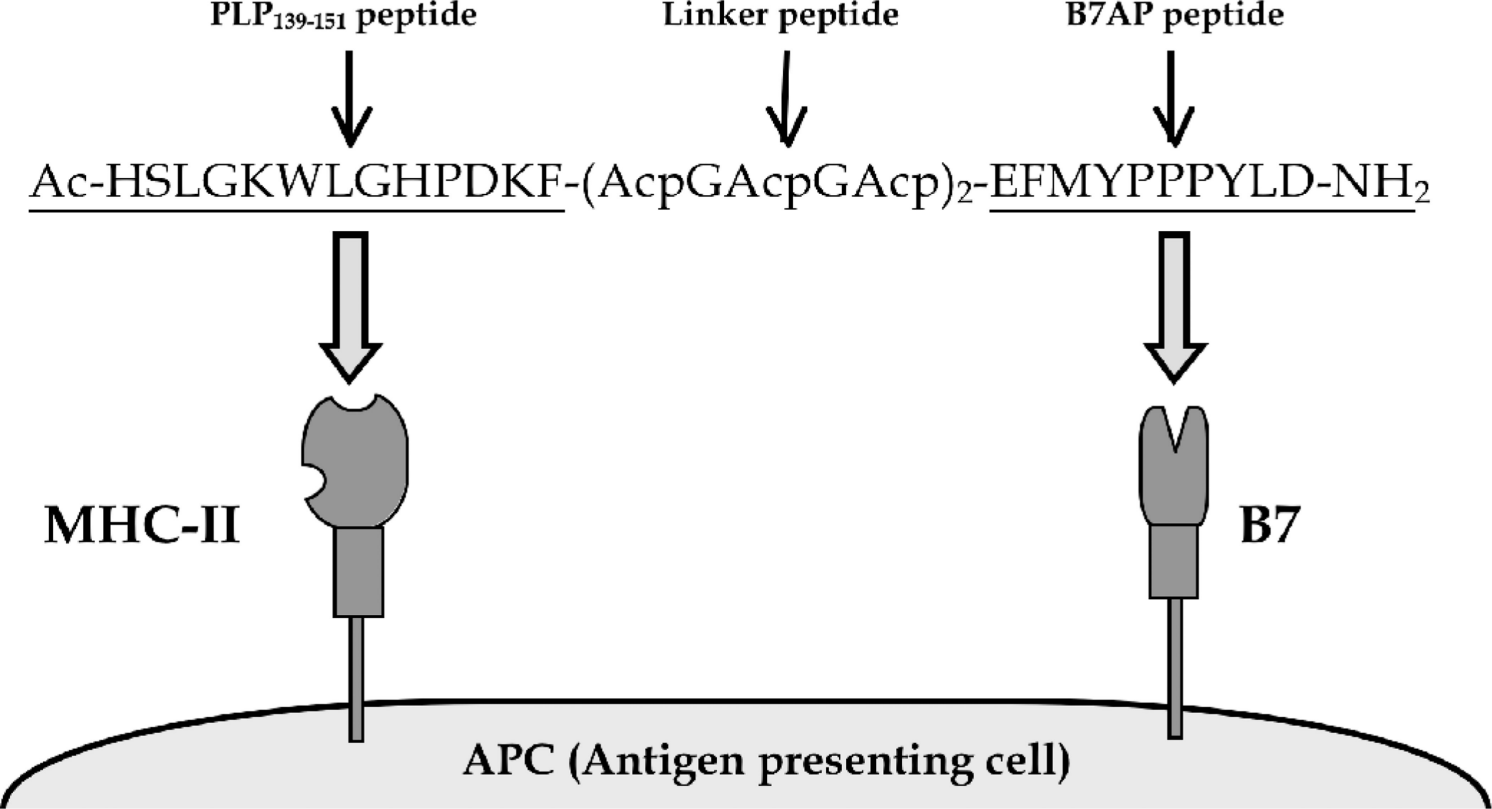
Sequence and target receptors of PLP-B7AP. PLP-B7AP is a linear 33-amino acid peptide composed of the antigenic peptide PLP_139–151_ and the B7 binding peptide B7AP, which is derived from the conserved region of the CD28 molecule. Both peptides are covalently conjugated to each other via a linker composed of ε-aminocaproic acid and glycine. The N- and C- termini of the peptide are capped by acetylation and amidation, respectively. The hypothesis is that the PLP_139–151_ portion will bind to MHC-II (I-A^s^), and B7AP will simultaneously bind to B7 on the surface of the APC.

In this study, B7AP was conjugated with an antigenic peptide (PLP_139–151_) derived from myelin proteolipid protein (PLP) to form a new BPI called PLP-B7AP (Figure 1). The hypothesis is that the PLP peptide portion of PLP-B7AP binds to empty MHC-II molecules and the B7AP portion binds to B7 on the surface of APC. In this case, PLP-B7AP tethers the two signals and prevents the complete segregation of Signal-1 and Signal-2 in the immunological synapse; thus altering T cell commitment from inflammatory to regulatory T cells [5]. In contrast to BFP which stimulates inflammatory response to treat cancer, PLP-B7AP alters the immune response via inhibition of pro-inflammatory T cells or proliferation of regulatory T cells to induce tolerance toward a specific antigen without suppressing the general immune response. To test the efficacy of PLP-B7AP, it was compared to several control peptides in suppressing EAE in the mouse model. The effect of PLP-B7AP peptide on cytokine production was evaluated to better understand the mechanism of action of PLP-B7AP in suppressing EAE.

## Material and Methods

### Mice

All protocols for experiments involving SJL/J (H-2s) mice (Charles River, Wilmington, MA) were approved by the University’s Institutional Animal Care and Use Committee. The mice were housed under specific pathogen-free conditions at a facility at The University of Kansas that is approved by the Association for Assessment and Accreditation of Laboratory Animal Care.

### Peptide synthesis

Peptides used in the present study are listed in Table 1. The peptides were synthesized using 9-fluorenylmethyloxy-carbonyl (Fmoc)-protected amino acid chemistry on a PEG-PS™ resin (Applied Biosystems, Foster City, CA) using a Pioneer™ peptide synthesizer (PerSeptiveBiosystems, Framingham, MA). Cleavage of the peptide from the resin and removal of the side-chain protecting groups were done using 90% TFA with 10% scavenger reagents (1,2-ethane dithiol (3%), anisole (2%), and thioanisole (5%)). After precipitation in ice-cold diethyl ether, the crude peptides were purified by reversed-phase HPLC using a semi-preparative C-18 column with a gradient of solvent A (94.9/0.1/5.0%=H_2_O/TFA/acetonitrile) and solvent B (100% acetonitrile). The purity and the identity of the peptides were confirmed using an analytical C-18 column and electrospray ionization mass spectrometry, respectively.

### Induction of EAE and clinical evaluation

An emulsion (0.2 ml) was prepared by mixing 0.1 ml of 200 µg PLP in phosphate-buffered saline (PBS) solution and 0.1 ml of complete Freund’s adjuvant (CFA) containing killed mycobacterium tuberculosis strain H37RA (Difco, Detroit, MI) with a final concentration of 4 mg/ ml. On day 0, the emulsion was subcutaneously (s.c.) injected into SJL/J female mice (5–7 weeks old) at regions above the shoulder and the flanks (total of 4 sites; 50 µl at each injection site). Then, 200 ng of pertussis toxin (List Biological Laboratories, Campbell, CA) was injected intraperitoneally (i.p.) on the day of immunization (day 0) and 48 h post-immunization. The clinical scores that reflect the disease progression were determined by the same observer in a blinded fashion using a scale ranging from 0 to 5 as previously described [26]. EAE clinical scores were evaluated as follows; 0-no clinical symptoms, 1-limp tail or waddling gait with tail tonicity, 2-waddling gait with limp tail (ataxia), 2.5-ataxia with partial paralysis of one limb, 3-full paralysis of one limb, 3.5-full paralysis of one limb with partial paralysis of the second limb, 4-full paralysis of two limbs, 4.5-full paralysis of two limbs with partial paralysis of forelimbs, and 5-moribund or dead. Body weight was also measured daily.

### *In vivo* peptide treatments

Study I:This study was performed to test the *In vivo* efficacy of PLP-B7AP in suppressing EAE. Mice were immunized on day 0 in order to develop EAE as described above. In our previous studies with other similar BPIs, we observed that a dosing regimen of 3 injections of BPI (100 nmol) on days 4, 7, and 10 were effective in prophylactic studies. Similarly, each mouse received s.c. injections of PLP-B7AP at a concentration of 100 nmol/100 µl/injection (in PBS) on days 4, 7, and 10. The efficacy of PLP-B7AP was compared to that of the vehicle (PBS), 100 nmol/100 µl of PLP, 100 nmol/100 µl of B7AP, and an equal mixture of PLP and B7AP (100 nmol each diluted in 100 µl PBS). The efficacy of each peptide was evaluated by monitoring the clinical score and the change in body weight over a period of 25 days.

Study II: The purpose of this study was to evaluate the potency of PLP-B7AP at a lower dose and lower frequency of injections. EAE was induced on day 0 as described above. The first group of mice received s.c. injections of PLP-B7AP at a concentration of 50 nmol/100 µl (in PBS) on days 4, 7, and 10, and its efficacy was compared to that of the negative control (100 µl PBS) and positive control (50 nmol/100 µl of PLP-BPI). In addition, another group of mice was treated with only one s.c. injection (100 nmol/100 µl) of PLP-B7AP on day 4. The potency of each treatment was evaluated using the clinical score and the change in body weight over a period of 25 days.

Study III: The efficacy of PLP-B7AP in a vaccine-like treatment was also evaluated, i.e., administration of peptide prior to induction of disease. In this study, the mice received three s.c. injections of PLP-B7AP (100 nmol/100 µl) on days –11, –8, and –5, and EAE was induced on day 0. The efficacy of PLP-B7AP when administrated prior to EAE induction was compared to that of the negative control (100 µl PBS). The efficacy of the peptide as a vaccine was evaluated by monitoring the clinical score and change in body weight over a period of 25 days.

### *In vitro* cytokine production

*In vitro* cytokine assays were performed following a protocol similar to that reported previously [30]. EAE was induced in SJL/J mice by injection of PLP/CFA and pertussis toxin as described above, and mice were treated with either PBS (100 µl) or PLP-B7AP (100 nmol/100 µl/ injection) on days 4, 7, and 10. Mice from the various treatment groups (n=3 per group) were sacrificed on the day of maximum disease (i.e., day 15) and day of remission (day 30) and their spleens were isolated. Single cell suspensions of splenocytes were harvested by gently mashing the spleen through a cell strainer using the rubber end of a 1-ml syringe in a petri dish containing serum-free RPMI-1640 supplemented with 10% fetal bovine serum, 100 Units of penicillin/100 µg streptomycin, 2 mM L-glutamine, and 50 µM 2-mercaptoethanol. Red blood cells were lysed using ACK lysis buffer (Invitrogen). The remaining splenocytes were then washed three times with serum-free RPMI-160 medium (Cellgro). The cells were then primed with PLP (20 µM) in a 24-well plate (5 × 10^6^ cells/well). Supernatants of cell cultures were collected for cytokine detection 72 h later and stored in a −80°C freezer until analysis. Secreted interleukin (IL)-2, IL-4, IL-5, IL-6, and IL-17 were measured by quantitative ELISA-based Q-PlexTM assay (Quansys Biosciences, Logan, UT).

### Histological analysis

EAE was induced in SJL/J mice by injection of PLP/CFA and pertussis toxin as described above, and mice were treated with either PBS (100 µl) or PLP-B7AP (100 nmol/100 µl/injection) on days 4, 7, and 10. On day 30, three mice from each treatment group were anesthetized and perfused transcardially with saline followed by ice-cold 4% paraformaldehyde in 0.1 M PBS (pH=7.4). Whole brains were harvested, sectioned in a sagittal manner, fixed in 4% paraformaldehyde for 48 h, and embedded in paraffin. Sagittal sections (5 µm thick) were stained with haematoxylin and eosin (H&E) or Luxol Fast Blue (LFB) and evaluated for infiltrating leukocytes and demyelination. All H&E histological scores were evaluated blindly by a board-certified pathologist (IHC World LLC, Woodstock, MD, USA). All LFB slides were analysed using the area fraction method. Stained tissue sections were examined under a Nikon Eclipse 90i light microscope and images were captured with a Nikon Digital Camera DXM 1200c and NIS Elements software (Nikon, Tokyo, Japan). Digital micrographs of the sections were obtained for each treatment group.

### Statistical analysis

Statistical analysis was done using one-way analysis of variance followed by Fisher’s least-significant difference to compare the different parameters, including EAE clinical scores, change in body weights, and *In vitro* cytokine production. All statistical analyses were performed using StatView software (SAS Institute, Inc., Cary, NC). A p-value of less than 0.05 was used as the criterion for statistical significance. All p-values for clinical scores and percent body weight changes were determined by comparing the data from day 10 through day 25.

## Results

### Study I: Suppression of EAE by PLP-B7AP

**Figure 2:**
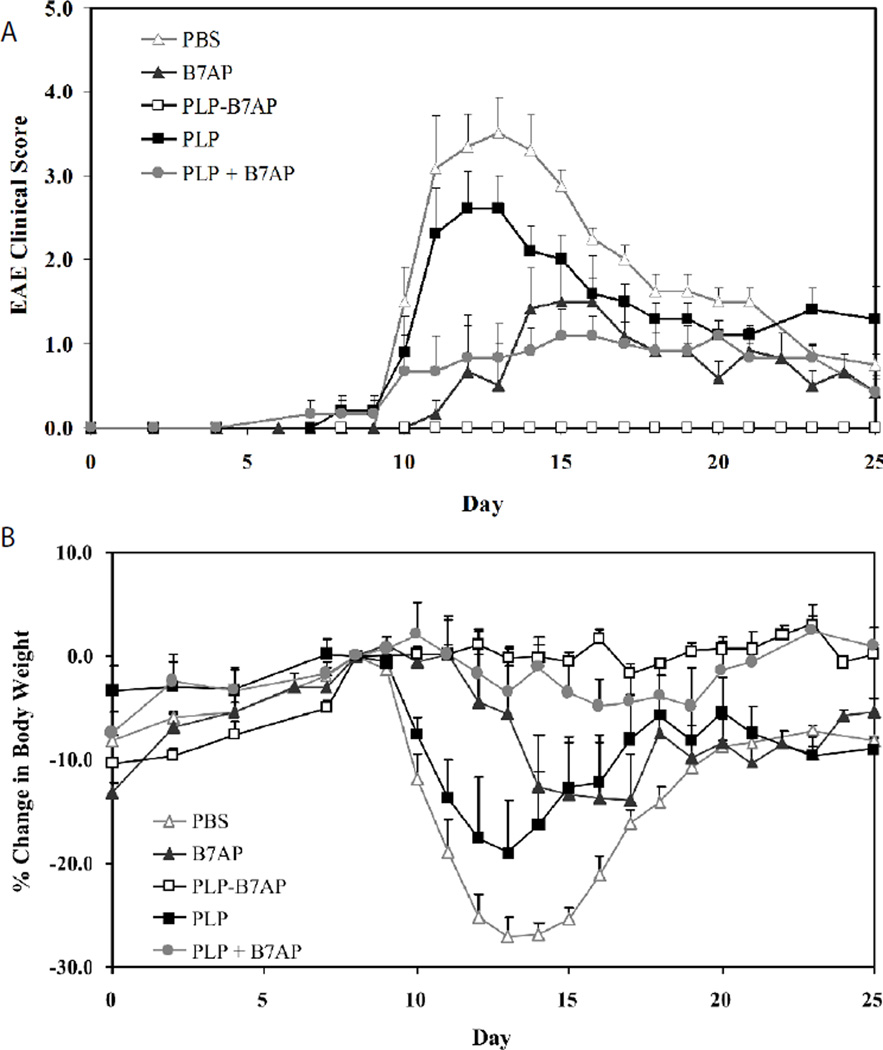
The *in vivo* efficacies of PLP-B7AP and controls in suppressing EAE in the mouse model determined by **(A)** clinical disease score and **(B)** percent change in body weight, relative to day 8 (day of disease onset). PBS-treated mice received s.c. injections of 100 µl of PBS on days 4, 7, and 10. PLP-, B7AP-, PLP + B7AP mixture-, and PLP-B7AP-treated mice received 100 nmol/100 µl in PBS on days 4, 7, and 10 (s.c.). Results are expressed as the mean clinical score ± SEM (*n* = 8 for PLP-B7AP and *n* = 6 for all others).

For the *in vivo* study I, the efficacy of PLP-B7AP in suppressing EAE was evaluated for the first time. Three injections were administered s.c. on days 4, 7, and 10. Its efficacy was compared to that of the vehicle (PBS) negative control as well as PLP, B7AP, and an unconjugated mixture of PLP and B7AP. Each peptide was administered at a concentration of 100 nmol/100 µl in a PBS solution. The clinical score results (Figure 2A) indicated that PLP-B7AP suppressed the disease completely with 100% of the mice remaining disease-free (p<0.0001 when compared to PBS treatment). All the PBS-treated mice exhibited severe signs of EAE, which peaked at day 13 with a maximal disease score of 3.5. The PLP-treated mice also exhibited severe signs of EAE, but it was still significantly less than the PBS-treated group (p<0.05), with a maximal disease score of 2.6. In addition, the B7AP peptide suppressed disease significantly when compared to PBS (p<0.0001) with a maximal score of 1.5, but it was still not as effective as PLP-B7AP (p<0.0001). Finally, to test the importance of the covalent linker connecting PLP to B7AP, the unconjugated mixture was also tested. It was found that it suppressed the disease slightly better than B7AP, but it was not significantly different (p>0.05) and was less effective than PLP-B7AP (p<0.0001). The loss in body weight correlated well with what was reported from the clinical scores (Figure 2B) except that the group treated with the PLP and B7AP mixture had significantly lower loss of body weight compared to the B7AP-treated mice (p<0.001). The PBS-treated mice lost approximately 27% of their body weight during the peak of the disease, while the PLP- and B7AP-treated mice lost 19% and 14% of their body weight, respectively. The mice treated with the unconjugated mixture of PLP and B7AP had a very small loss in body weight with a maximum of 4.9%, while most of the PLP-B7AP-treated mice exhibited a gain in body weight, resulting in a maximal loss of 1.6% for the group.

### Study II: Potency of PLP-B7AP

**Figure 3:**
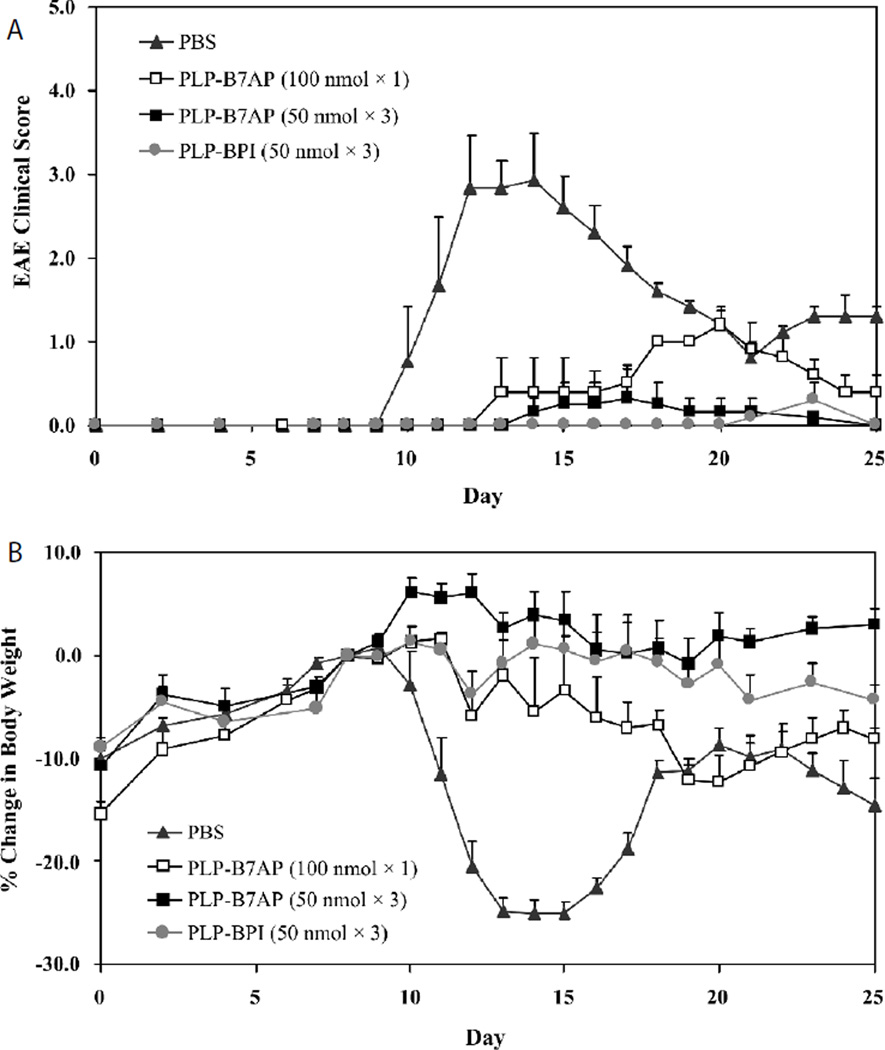
The *in vivo* dose-dependency and potency of PLP-B7AP in suppressing EAE in the mouse model as determined by **(A)** clinical disease score and **(B)** percent change in body weight, relative to day 8 (day of disease onset). PLP-BPI was used as a positive control. The mice were treated with the peptides after immunization with PLP/CFA on day 0. For the negative control, mice received s.c. injections of 100 µl of PBS on days 4, 7, and 10. One group received one s.c. dose of 100 nmol/100 µl in PBS on day 4 of PLP-B7AP treatment. The remaining groups received three s.c. injections of PLP-B7AP and PLP-BPI (positive control) at a dose of 50 nmol/100 µl in PBS on days 4, 7, and 10. The efficacy and potency of the peptide were determined by clinical disease score of EAE. Results are expressed as the mean clinical score ± SEM (*n* = 6).

In the second *in vivo* study, the potency of PLP-B7AP was evaluated at a lower dose, reduced dosing frequency, and compared to that of a previously well-studied BPI molecule, PLP-BPI [28,29,31]. First, the dose was lowered from 100 to 50 nmol while maintaining three injections. Second, the dose was kept at 100 nmol but the dosing schedule was changed to only one injection. As shown by the results in Fig 3A, a 50 nmol dose of PLP-B7AP administered three times was not as effective as 100 nmol dose administered three times (Figure 2A). At a higher dose, PLP-B7AP completely suppressed the disease with all the animals showing no symptoms of the disease. Whereas when the dose was reduced by half, we observed that some animals did exhibit symptoms of EAE with a clinical score as high as 1.2 (Figure 3A) and percent loss in body weight as high as 12.2% (Figure 3B). Similarly, one injection was even less effective in suppressing the progression of the disease. Nevertheless, PLP-B7AP was better at a lower dose and suppressed the disease more efficiently compared to the PBS-treated mice (p<0.0001). Also, the efficacy of one injection (50 nmol/100 µl) of PLP-B7AP was compared to that of one injection of PLP-BPI (50nmol/100 µl). The clinical score (Figure 3A) results indicated that there was no significant difference between the PLP-B7AP- and the PLP-BPI-treated mice (p>0.05). The average maximum clinical scores that the PLP-B7AP- and the PLP-BPI-treated mice reached were 0.33 and 0.3, respectively. Both treatments suppressed disease significantly when compared to PBS treatment (p<0.0001). However, there was a significant difference in the loss or gain of body weight (Figure 3B) between the PLP-B7AP- and PLP-BPI-treated mice (p<0.05). PLP-BPI treated mice lost a small amount of body weight, reaching a maximum of 4.36%, while the PLP-B7AP-treated group had a significantly healthier gain in body weight.

### Study III: Vaccination with PLP-B7AP

**Figure 4:**
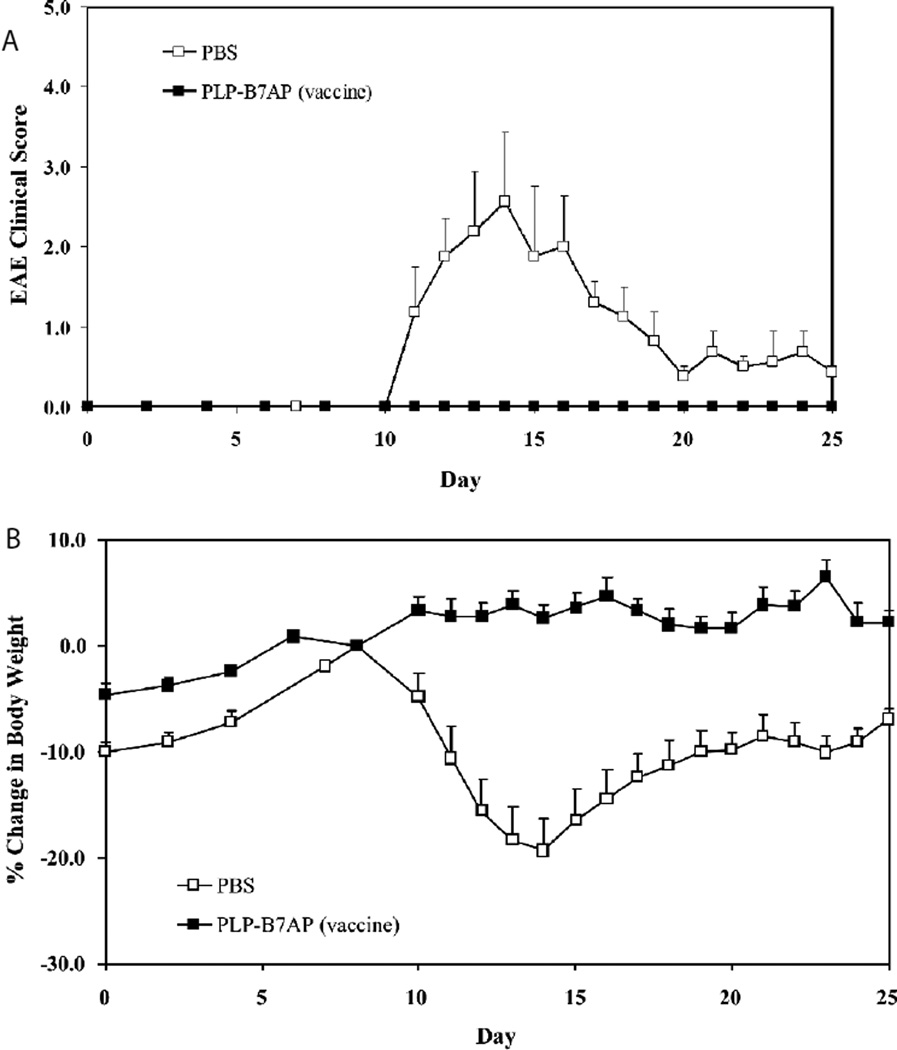
Evaluation of the *in vivo* efficacy of PLP-B7AP as a vaccine in suppressing EAE as determined using **(A)** clinical disease score of EAE and **(B)** percent change in body weight, relative to day 8 (day of disease onset). The negative control is the PBS-treated group, which received s.c. injections of 100 µl of PBS on days −11, −8, and −5. The PLP-B7AP group was vaccinated on days −11, −8, and −5. Each mouse received a s.c. injection of 100 nmol/100 µl in PBS. Results are expressed as the mean clinical score ± SEM (*n* = 6).

For the final *in vivo* study, the hypothesis was that BPI molecules act by promoting the regulatory immune response toward the specific antigen in the BPI molecules. Therefore, 100 nmol/injection of PLP-B7AP was injected on days −11, −8, and −5, which is prior to induction of disease. According to the clinical score, PLP-B7AP suppressed EAE completely in a fashion similar to that when it was injected after the induction of disease. In contrast, the PBS-treated mice reached a maximum clinical score of 2.56 (Figure 4A). The change in body weight of the mice confirmed the results shown by the clinical score, since again in body weight was observed only in the PLP-B7AP-treated mice, and the PBS-treated mice lost a maximum of 19.4% (Figure 4B).

### *In vitro* cytokine production

To better understand the mechanism of action of PLP-B7AP, splenocytes were isolated and their cytokine production was determined using a quantitative ELISA-based Q-PlexTM assay. This method unfortunately does not determine exact concentrations of circulating cytokines, but does provide information regarding the general immune response in the body. If there is a general inflammatory response, we would expect to see more Th17 and Th1 phenotypes, both of which are crucial players in the progression of EAE. To treat EAE, therapies need to promote the regulatory and suppressor T-cell phenotypes (i.e., more production of T reg and Th2). The prevalent phenotype of T cells can be determined by the cytokines produced by the splenocytes. If there is a higher population of Th1 and Th17 cells, there will be a greater production of pro-inflammatory cytokines such as IL-6 and IL- 17. Anti-inflammatory cytokines (IL-2, IL-4, and IL-5) either suppress inflammatory responses or promote regulatory responses, and therefore a greater concentration of these cytokines indicates a suppressor or regulatory immune response.

**Figure 5:**
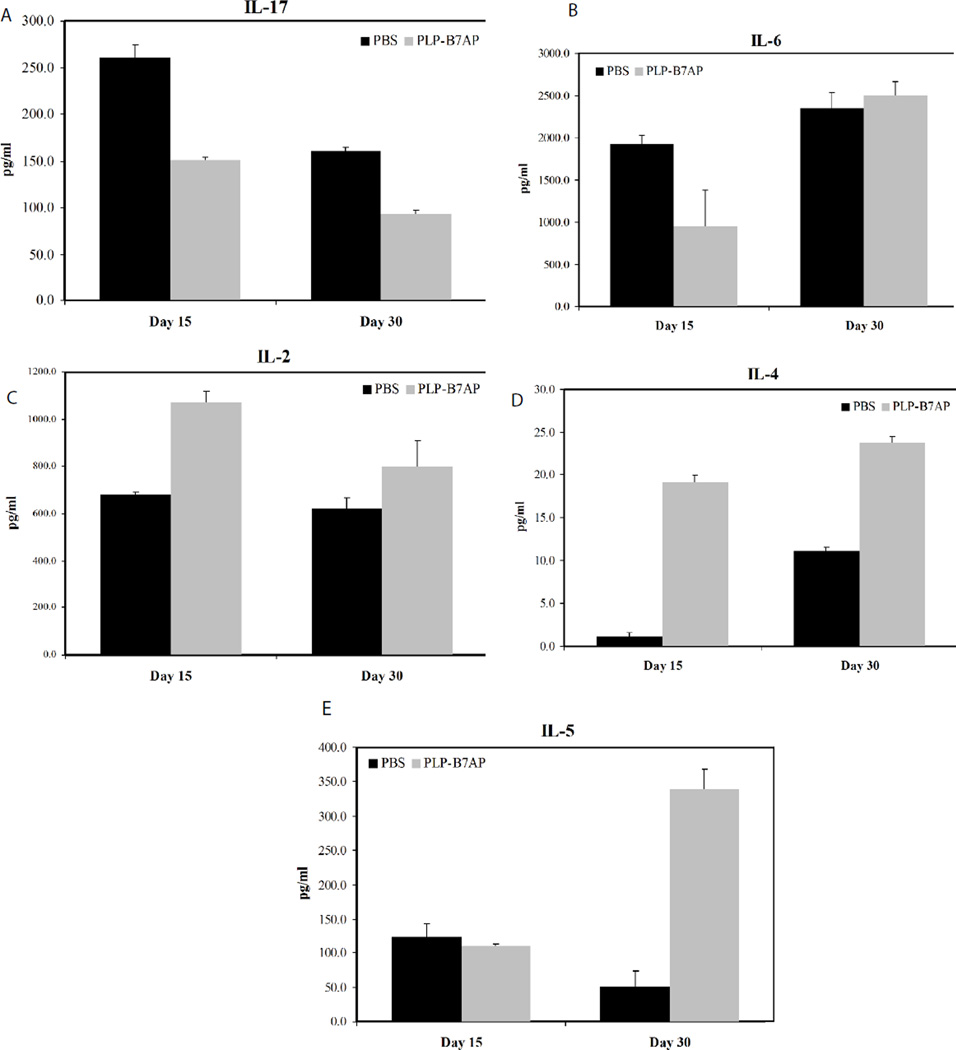
Concentrations of the secreted cytokines from the cell culture supernatant: **(A)** IL-17, **(B)** IL-6 (C) IL-2, (D) IL-4, and (E) IL-5. Splenocytes were isolated from the spleens of EAE-induced mice on either day 15 or 30. Each mouse was treated with either PBS or PLP-B7AP on days 4, 7, and 10. The pooled splenocytes (*n* = 3 mice) were stimulated *in vitro* with PLP_139–151_, and supernatant was isolated 72 h later for cytokine detection. Statistical values were as follows: *p* < 0.0001 for IL-17, *p* < 0.05 for IL-6, *p* < 0.0001 for IL-2 and IL-4; *p* < 0.001 for IL-5.

As described in the Materials and Methods section, *in vitro* cytokine analysis was designed following a similar protocol to that outlined in study I. After induction and treatment, splenocytes were isolated on days 15 and 30, which correspond to the day of most severe disease and the day when EAE is in remission, respectively, and their cytokine production was measured to determine the T-cell phenotype present. For the PBS group, the average EAE clinical scores were 2.88 ± 0.48 and 0.90 ± 0.55 on days 15 and 30, respectively. In contrast, PLP-B7AP-treated mice did not shown EAE symptoms and their scores were zero throughout the study. The cytokine production from splenocytes harvested from naïve mice was not evaluated. However, *In vitro* proliferation of splenocytes isolated from naïve mice and treated with PLP was significantly lower compared to splenocytes isolated from previously EAE-induced mice [24, 28]. This indicates that the immune response from naïve mice treated with PLP would be minimal, and therefore, cytokine production would be minimal or probably undetectable. On the other hand, there was a significantly higher proliferation of splenocytes in EAE-induced mice treated with PBS compared to those treated with BPI [24,29]. This implies that there was an expansion of PLP-specific T cells in untreated mice compared to BPI treated mice. At the day of maximum disease score (day 15), there was a significant drop in the production of IL-17 in the PLP-B7AP-treated mice compared to the PBS-treated mice (p<0. 0001, Figure 5A). At day 30, PLP-B7AP-treated mice produced a much lower amount of IL-17 compared to the PBS-treated mice, (p<0.0001). This is probably due to the fact that, at this stage, the disease is much weaker. Another pro-inflammatory cytokine tested was IL-6, which contributes to Th17 and Th1 differentiation. On day 15, the production of IL-6 in the PLP-B7AP-treated mice was lower than in the PBS-treated mice (p<0.05, Figure 5B). At day 30, there was no difference in the production of IL-6 in the PBS- and PLP-B7AP-treated mice. There was a significant change in IL-6 concentration on the day of maximum disease but not after disease remission (Figure 5B), suggesting that the suppression of IL-6 is most critical during the development of the disease.

To monitor whether PLP-B7AP influences the regulatory/suppressor immune response, production of anti-inflammatory cytokines was measured. Although IL-2 cannot be linked directly to T reg, IL-2 has a major role in the growth and maintenance of T reg, and is important for maintaining immunological tolerance [32–35]. Therefore, IL-2 production was monitored after treatment of mice with PLP-B7AP. On both days 15 and 30, there was an increase in the production of IL-2 by the splenocytes from PLP-B7AP-treated mice compared to those from PBS-treated mice (p<0.0001 for day 15 and p=0.06 for day 30, Figure 5C). For the key Th2 cytokine markers (i.e., IL-4 and IL-5), PLP-B7AP-treated mice produced a significantly greater amount of IL-4 on both days 15 and 30 (p<0.0001, Figure 5D) compared to PBS-treated mice. However, the production of IL-5 was significantly higher only on day 30 for PLP-B7AP-treated mice compared to PBS-treated mice (p<0.001); there was no observable difference in IL-5 production on day 15 for either PLP-B7AP- or PBS-treated mice (Figure 5E). These results indicate that PLP-B7AP treatment promotes an immunological tolerant state.

### Histological analysis of brain sections

To evaluate whether treatment of EAE with BPI addressed other aspects of the disease, we conducted histological analysis of the brain tissue at the end of the study (day 30) to determine whether we were able to decrease infiltration of inflammatory cells into the CNS and demyelination. H&E staining did not show any significant difference in leukocyte infiltration. All the brains sections in both groups showed perivascular inflammation and moderate infiltration of the cerebellar white matter. It is possible that we did not observe any difference in infiltrated leukocytes between the two groups because the disease could probably be in remission. Accumulated evidence indicate that with time, as MS progresses it enters a secondary progressive disease phase that is usually characterized by decreased inflammatory activity but more frequent relapses [36].

**Figure 6:**
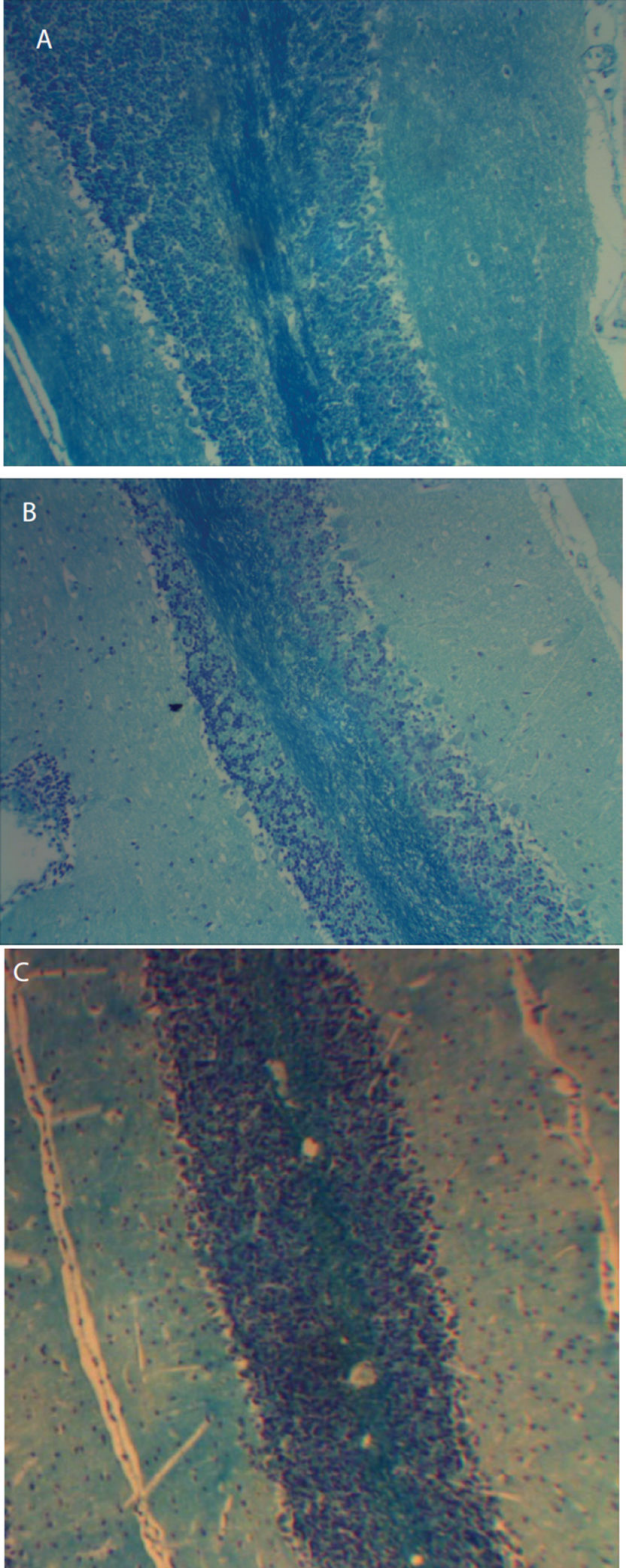
Histological analysis of cortical brain sections isolated from mice induced with EAE and treated with either (A) PLP-B7AP, or (B) PBS. Cortical brain sections isolated from naïve mice (C) were also evaluated. Cortical brain sections were stained with Luxol Fast Blue in order to evaluate for demyelination in the CNS. Brain sections isolated from PLP-B7AP treated mice had significantly (*p* < 0.0001) demyelination than PBS treated mice.

LFB staining of cortical brain sections were used to evaluate axonal injury and possibly correlate it to formation of lesions. Sectioning and staining were evaluated blindly and commercially to avoid any bias. Also, these cortical sections were evaluated when the disease had progressed possibly into a secondary phase and these sections should provide a good representation to other regions of the brain such as the spinal cord. Although EAE is an ascending disease, we expect to have a diffuse in axonal injury throughout the whole brain and the spinal cord during the secondary disease phase. Our results showed that the control group had significantly higher demyelination compared to BPI treated mice (Figure 6A and B). Area fraction analysis in brain sections stained with LFB showed that myelin covered area BPI and control groups were calculated to be 24.3% (Figure 6A) and 9.7% (Figure 6B), respectively; suggesting that there was less myelin in the control group due to extensive demyelination. Naïve mice had about 28.3% myelination (Figure 6C) and were statistically similar to percent myelination in PLP-B7AP group; further indicating that PLP-B7AP was effective in inhibiting inflammation in the CNS.

## Discussion

Here, the efficacy of the most recently developed BPI molecule is reported for the first time, and all the *In vivo* results from the present study are summarized in Table 2. PLP-B7AP has efficacy similar to that of the previously studied molecule known as PLP-BPI [28,29,31]. In this study, our results showed that PLP-B7AP was significantly better than unconjugated PLP or B7AP at delaying the onset of EAE and suppressing it. More importantly, PLP-B7AP treatment was more effective than that of a mixture of unconjugated PLP and B7AP, indicating that the conjugation has an important role in the mechanism of action. Numerous reports have shown that soluble antigenic peptides can induce immune tolerance [37]. It is suggested that they can bind directly to dendritic cells in lymphoid tissues and induce differentiation of naïve cells into regulatory T cells. Similarly, our results showed that PLP or a mixture of PLP and B7AP had some EAE-suppressive activity because they contain soluble antigenic peptides. It is also possible that PLP or PLP-B7AP could induce anergy of the T cells. However, this does not explain why PLP-B7AP is more potent than PLP or a physical mixture of PLP and B7AP. We can speculate that BPIs including PLP-B7AP are more potent because they can simultaneously target the two signals involved in an immune response. BPI tethers the two signals and inhibits the formation of the immunological synapse thus altering the differentiation of naïve T cells to regulatory T cells while inhibiting activation of pro-inflammatory T cells. Also, it was interesting to find that when PLP-B7AP was injected prior to induction of disease (vaccine treatment), EAE was completely suppressed as seen by the clinical score and normal increase in body weight. It is well known that peptides usually have a short half-life and short residence time in the systemic circulation; however, PLP-B7AP was still effective when delivered in a vaccine dose schedule where the last injection was 5 days before disease stimulation (day 0). This result suggests that a potential mechanism of PLP-B7AP is that it could stimulate the regulatory immune response (i.e., an increased production of T regs) prior to disease stimulation. Thus, when EAE was induced, T regs responded by downregulating any Th1- and Th17-mediated inflammatory response toward the antigen and, therefore, prevented the onset of the disease.

The B7AP portion of PLP-B7AP was derived from CD28 [22], which bind to the B7 molecule and can hinder the activation of B7/ CD28 costimulatory signal [7,8]. Another important molecule called CTLA-4 also binds to B7. The CTLA-4/B7 interaction provides an inhibitory signal and thus prevents T-cell activation [13]. Therefore, this signal is important for down regulating an undesirable inflammatory response in MS or EAE. However, due to the relatively fast binding kinetics of B7 and CTLA-4 [38] and the high avidity of this interaction [39,40], it was proposed that B7AP would not affect this interaction [22]. As a result, B7AP selectively inhibits the CD28/B7 signal while not affecting the CTLA-4/B7 signal. The other major portion of the peptide is PLP, which binds to the MHC-II molecule on the surface of APC. The third portion of the peptide is the covalent linker, which connects PLP and B7AP. The linker is a vital part of the peptide and may provide two advantages. The first advantage of linking the two peptides is that it allows the blockade of Signal-2 only in T cells that recognize the PLP portion of the peptide as antigenic, leading to specific immunomodulation. It is known that specificity is a major problem in most therapies aimed at attenuating the immune response. Due to the lack of specific immunosuppression, the patient can become susceptible to opportunistic infections due to suppression of the general immune response, which is the case with most current therapeutic agents. The second proposed advantage is that the two peptide fragments can bind to their respective receptors on the surface of the APC, thus tethering them together and preventing the formation of the immunological synapse. This may alter the differentiation of T cells from inflammatory to regulatory cells.

Previously, several BPI molecules that target other APC surface receptors, such as PLP-BPI and PLP-cIBR, have been developed to bind the adhesion molecules ICAM-1 and LFA-1, respectively [5,25,28,29,31]. Both of these adhesion molecules have crucial roles in the activation of T cells, especially after formation of the immunological synapse [4,41,42]. The mechanism of action of these peptides is not yet well understood, but it is hypothesized that they act by hindering the formation of the immunological synapse. We have previously investigated the binding of another BPI (i.e., GAD-BPI) to I-Ag^7^ and ICAM-1 on the surface of APC [43]. We observed a significantly higher co-localization of I-Ag^7^ and ICAM-1 receptors on the surface of GAD-BPI-treated APC compared with APC treated with a mixture of unlinked GAD and LABL peptides; suggesting that GAD-BPI can bridge I-Ag^7^ and ICAM-1 on the surface of APC. There was a significantly higher percentage of cells with higher co-localization of I-Ag^7^ and ICAM-1 receptors in GAD-BPI-treated cells compared to these treated with unlinked peptides. This further suggests that GAD-BPI can tether the two signals and inhibit the formation of the immunological synapse. Furthermore, co-localization was blocked by either anti-I-Ag^7^ or anti- ICAM-1 mAbs [43]; indicating that BPI can bind to I-Ag^7^ and ICAM-1. This result supports the possible mechanism of action of BPI molecules, which is to prevent immunological synapse formation. Although the binding properties of PLP-B7AP to both B7 and MHC-II have not been determined yet, it is expected PLP-B7AP to bind these receptor in a similar manner. In future, the activity of different BPI molecules (i.e., PLP-BPI, PLP-cIBR, or PLP-B7AP) to induce co-localization of MHC-II and the respective co-stimulatory molecule (i.e., ICAM-1, LFA-1, or CD28) on different APC (i.e., B cells, dendritic cells, and macrophages) will be investigated. In the present study, there was no difference (p>0.05) in efficacy between PLP-B7AP and PLP-BPI, which target B7 and ICAM-1, respectively. This could be due to the fact that both molecules are expressed on the surface of APC and are up regulated when the APC are activated. Another important mechanism, which may explain the similar efficacies, could be due solely to the antigenic peptide portion, with the adhesion peptide and B7-peptide acting as targeting molecules. Antigenic peptides have become very useful in the treatment of autoimmune and allergic diseases. The proposed mechanism of action for soluble antigenic peptides is that they bind directly to empty MHC-II molecules on the surface of naive APC such as immature dendritic cells (iDC) [37]. The presentation of an antigen by an iDC without any antigen processing and in the absence of a co-stimulatory signal is believed to lead to differentiation of naïve T cells to regulatory T cells. Therefore, another proposed mechanism of action is that BPI molecules can bind to iDC, resulting in activation of the regulatory immune response and inducing antigen-specific tolerance.

Although the mechanism of action of BPI molecules is not yet fully known, the results of the cytokine profiles from the current study and other studies conducted by our laboratory provide clues on how these peptides modulate the immune response. In the current study, the results indicate that treatment with PLP-B7AP lowers the production of IL-17 on days 15 and 30. This indicates a down-regulation of Th17 cells, a crucial player in the pathogenesis of EAE [44,45]. These results correlate with previous studies using other BPI molecules, such as PLP-BPI [26–28] and PLP-cIBR [25]. In addition, treatment with PLP-B7AP lowered the production of a pro-inflammatory cytokine IL-6 on day 15. It has been reported that blocking IL-6R lowers differentiation of Th17 and Th1 [46]. Therefore, the reduction in IL-6 on day 15 with PLP-B7AP treatment may provide similar suppression and protect against EAE. There was also an increase in the production of IL-2 and IL-4 on days 15 and 30 and IL-5 on day 30 only. Increased production of IL-2 promotes immunological tolerance and ameliorates autoimmunity by stimulating T regs growth and survival [32–35], while more IL-4 and IL-5 indicates a shift towards a Th2 phenotype, which is immunosuppressive in MS and EAE [21]. Previously, we have demonstrated that other BPI molecules promote T regs and Th2 phenotype as indicated by the proliferation of CD4+CD25+IL10+ and CD4+CD25+IL4+ upon PLP-BPI treatment [29]. In addition, *in vitro* cytokine studies indicated that PLP-cIBR stimulates production of IL-4 as well as IL-10 and TGF-β, which High enhancement of the Gd-DTPA signal was observed in the non-treated are important for T regs growth and function [47–49]. Therefore, the data from this study, as well as other studies conducted by our laboratory, provide us with some evidence that BPI molecules can modulate the immune response by shifting the balance away from the inflammatory to an immunotolerant state.

CNS inflammation and leukocyte infiltration are major aspects in the pathogenesis of MS and EAE due to their role in BBB breakdown and demyelination. As mentioned above, the infiltration of inflammatory cells into the CNS has been determined using H&E staining of cortical brain sections isolated on day 30. However, H&E did not show any significant difference in inflammatory infiltrates and inflammatory cuffs in the two groups. It is possible that on day 30, the disease had progressed into a secondary phase characterized by less inflammatory activity. In most autoimmune diseases including MS, more intense inflammation is prevalent during the initial phase whereas the secondary and progressive phase is usually characterized by reduced inflammatory activity [36]. Pathology and MRI studies in MS patients have shown that inflammation is prevalent in early and active lesions but absent in late MS [50]. It has been suggested that the lack of significant lymphocyte inflammation particularly in the case of extensive subpial lesions implies that substances present in the cerebrospinal fluid maybe the mediators of axonal injury [51]. Our cytokine data demonstrate that BPI molecules are capable of down-regulating the inflammatory response peripherally and we conducted studies that directly examined the effects of BPI on the CNS. Using magnetic resonance imaging (MRI), BBB integrity can be evaluated by measuring CNS deposition of gadolinium diethylenetriaminepentaacetate (Gd-DTPA). High enhancement of the Gd-DTPA signal was observed in the non-treated but not in the normal mice (no EAE) and in the PLP-BPI-treated mice [26]. In addition, histological analysis of the brains of mice treated with another BPI molecule, PLP-cIBR, indicated that treatment significantly prevented demyelination in the CNS [25]. Similarly, histological analysis showed that PLP-B7AP inhibited demyelination; indicating that like all our other BPI molecules PLP-B7AP has CNS protective effects that prevent the progression of disease in mice.

Previously, the pharmacokinetics (PK) profile of intravenously (i.v.) administered PLP-BPI in Sprague-Dawley rats showed that PLP-BPI is cleared rapidly with a half-life of about 2.2–3.5 hrs [27]. The s.c. injections of PLP-BPI in solution and microparticle controlled-release showed that both this route and delivery method are more effective than the intravenous injections [31]. It is expected that PLP-B7AP has a PK profile similar to that of the PLP-BPI. However, further pharmacokinetic and pharmacodynamic studies need to be performed to optimize the efficacy of BPI molecules and to translate their use to the clinic. In addition, PLP-B7AP has not been tested to reverse EAE after the disease has been induced. Previously, it has been shown that mice receiving three injections (100 nmol/injection) of PLP-BPI after developing EAE with a disease score of 1 or higher established remission significantly faster than non-treated (PBS) mice and did not undergo disease relapse as did the non-treated mice [28]. In the future, we hope to perform a compressive pharmacokinetic evaluation of BPI molecules and modify the treatment regimen (i.e., administer after signs of disease) to better match the actual clinic setting.

While there is still some skepticism concerning the use of animal models to test therapies for MS [52], several of the current drug candidates in clinical trials were initially investigated using these models. It is true that the disease pathogeneses of EAE and MS are different, but the underlying mechanisms and resulting symptoms are similar. Both diseases exhibit an inflammatory response initiated by CD4+ T cells. Once the knowledge of how to modulate the immune response to specific proteins in animal models has been developed, it may become possible to translate this to humans. The potential application for this specific immunomodulation is enormous. Many clinical trials are already underway to try to block Signal-2 molecules for the treatment of autoimmune diseases and allograft rejection. Unfortunately, solely blocking Signal-2 has proven to be dangerous, and many therapies have failed due to the onset of severe side effects from general immunosuppression. In the present study, a peptide that specifically targets T cells that recognize PLP as antigenic while simultaneously blocking the delivery of Signal-2 has been developed and may be used one day for antigen-specific immunosuppression. This form of treatment has been very successful in the mouse model of EAE, and can potentially be a safe way to attenuate the immunogenic response in MS without suppressing the general immune response. Moreover, other antigens can be conjugated to these Signal-2 blockers in order to treat epitope-spreading problems seen in MS. More importantly, this technology can potentially be translated to the treatment of any other form of autoimmune or allergic disease in which the antigens are known. Our laboratory has developed other BPI molecules such as GAD-BPI and CII-BPI with different antigenic peptides for treating different autoimmune diseases in animal models. GAD-BPI suppresses type-I diabetes in the non-obese diabetes mouse model [43] and CII-BPI suppresses rheumatoid arthritis in the collagen-induced arthritis mouse model. Once there is a better understanding of the mechanism of action of these peptides, these same strategies may be employed for the treatment of human immune diseases.

In conclusion, PLP-B7AP, when administered either before or after the induction of the disease, has been shown to be effective in suppressing EAE. Although the investigation of PLP-B7AP in the EAE animal model may not translate directly to humans, this study could improve our understanding of how to effectively modulate the immune response in an antigen-specific manner. The hope is that BPI molecules such as PLP-B7AP could be used in treating autoimmune diseases without suppressing the general immune response.

## Figures and Tables

**Table 1:** List of peptides used in the present study.

Peptide	Sequence
PLP_139–151_	HSLGKWLGHPDKF
B7AP	Ac-EFMYPPPYLD-NH_2_
PLP-B7AP	Ac-HSLGKWLGHPDKF-(AcpGAcpGAcp)_2_-EFMYPPPYLD-NH_2_
PLP-BPI	Ac-HSLGKWLGHPDKF-(AcpGAcpGAcp)_2_-ITDGEATDSG-NH_2_

Acp in the linker represents ε-aminocaproic acid. Ac- represents the acetyl-capped N-terminus of the peptide. −NH_2_ represents the amide-capped C-terminus of the peptide.

**Table 2:** Summary of *In vivo* studies.

Group	Dose^a^	Disease incidence^b^	Mean maximal score ± SEM
*In vivo* Study I: Preventive Treatment			
PBS	100 µl/mouse on days 4, 7, and 10	100%	3.50 ± 0.43
PLP	100 nmol/mouse on days 4, 7, and 10	100%	2.60 ± 0.44
B7AP	100 nmol/mouse on days 4, 7, and 10	83%	1.50 ± 0.55
PLP + B7AP	100 nmol/mouse on days 4, 7, and 10	100%	1.08 ± 0.33
PLP-B7AP	100 nmol/mouse on days 4, 7, and 10	0%	0.00 ± 0.00
*In vivo* Study II: Potency and Dose Dependency			
PBS	100 µl/mouse on days 4, 7, and 10	100%	2.92 ± 0.55
PLP-B7AP	100 nmol/mouse on day 4	100%	1.20 ± 0.20
PLP-B7AP	50 nmol/mouse on days 4, 7, and 10	17%	0.33 ± 0.33
PLP-BPI	50 nmol/mouse on days 4, 7, and 10	17%	0.30 ± 0.20
*In vivo* Study III: Vaccination Treatment			
PBS	100 µl/mouse on days –11, –8, and –5	100%	2.56 ± 0.88
PLP-B7AP	100 nmol/mouse on days –11, −8, and −5	0%	0.00 ± 0.00

a All injections were administered subcutaneously.

b Incidence of disease was defined as a disease score of 1 or higher.

## References

[1] Lassmann H (2001). Classification of demyelinating diseases at the interface between etiology and pathogenesis. Curr Opin Neurology.

[2] Badawi AH, Siahaan TJ (2012). Immune modulating peptides for the treatment and suppression of multiple sclerosis. Clinical Immunol.

[3] Grakoui A, Bromley SK, Sumen C, Davis MM, Shaw AS (1999). The immunological synapse: a molecular machine controlling T cell activation. Science.

[4] Tseng SY, Dustin ML (2002). T-cell activation: a multidimensional signaling network. Cur Opin Cell Biol.

[5] Manikwar P, Kiptoo P, Badawi AH, Buyuktimkin B, Siahaan TJ (2012). Antigen-specific blocking of CD4-specific immunological synapse formation using BPI and current therapies for autoimmune diseases. Med Res Rev.

[6] Chittasupho C, Siahaan TJ, Vines CM, Berkland C (2011). Autoimmune therapies targeting costimulation and emerging trends in multivalent therapeutics. Therapeutic Delivery.

[7] Jenkins MK, Johnson JG (1993). Molecules involved in T-cell costimulation. Curr Opin Immunol.

[8] June CH, Bluestone JA, Nadler LM, Thompson CB (1994). The B7 and CD28 receptor families. Immunology Today.

[9] Peach RJ, Bajorath J, Brady W, Leytze G, Greene J (1994). Complementarity determining region 1 (CDR1)- and CDR3-analogous regions in CTLA-4 and CD28 determine the binding to B7-1. J Exp Med.

[10] Truneh A, Reddy M, Ryan P, Lyn SD, Eichman C (1996). Differential recognition by CD28 of its cognate counter receptors CD80 (B7.1) and B70 (B7.2): analysis by site directed mutagenesis. Molecular Immunol.

[11] Bajorath J, Metzler WJ, Linsley PS (1997). Molecular modeling of CD28 and three-dimensional analysis of residue conservation in the CD28/CD152 family. J Mol Graph Model.

[12] Blanco B, Holliger P, Alvarez-Vallina L (2002). Autocrine costimulation: tumor-specific CD28-mediated costimulation of T cells by in situ production of a bifunctional B7-anti-CEA diabody fusion protein. Cancer Gene Ther.

[13] McCoy KD, Le Gros G (1999). The role of CTLA-4 in the regulation of T cell immune responses. Immunol Cell Biol.

[14] Schonbeck U, Libby P (2001). The CD40/CD154 receptor/ligand dyad. Cell Mol Life Sci.

[15] Valitutti S, Dessing M, Aktories K, Gallati H, Lanzavecchia A (1995). Sustained signaling leading to T cell activation results from prolonged T cell receptor occupancy. Role of T cell actin cytoskeleton. J Exp Med.

[16] Khoury S, Sayegh MH, Turka LA (1999). Blocking costimulatory signals to induce transplantation tolerance and prevent autoimmune disease. Int Rev Immunol.

[17] Podojil JR, Miller SD (2009). Molecular mechanisms of T-cell receptor and costimulatory molecule ligation/blockade in autoimmune disease therapy. Immunol Rev.

[18] Wetzig R, Hanson DG, Miller SD, Claman HN (1979). Binding of ovalbumin to mouse spleen cells with and without carbodiimide. J Immunol Methods.

[19] Jenkins MK, Mueller D, Schwartz RH, Carding S, Bottomley K (1991). Induction and maintenance of anergy in mature T cells. Adv Exp Med Biol.

[20] Jenkins MK, Schwartz RH (1987). Antigen presentation by chemically modified splenocytes induces antigen-specific T cell unresponsiveness in vitro and in vivo. J Exp Med.

[21] Miller SD, Turley DM, Podojil JR (2007). Antigen-specific tolerance strategies for the prevention and treatment of autoimmune disease. Nat Rev Immunol.

[22] Chen J, He Q, Zhang R, Chu Y, Wang Y (2004). Allogenic donor splenocytes pretreated with antisense peptide against B7 prolong cardiac allograft survival. Clin Exp Immunol.

[23] Srinivasan M, Gienapp IE, Stuckman SS, Rogers CJ, Jewell SD (2002). Suppression of experimental autoimmune encephalomyelitis using peptide mimics of CD28. J Immunol.

[24] Badawi AH, Siahaan TJ (2013). Suppression of MOG- and PLP-induced experimental autoimmune encephalomyelitis using a novel multivalent bifunctional peptide inhibitor. J Neuroimmunol.

[25] Kiptoo P, Buyuktimkin B, Badawi AH, Stewart J, Ridwan R (2013). Controlling immune response and demyelination using highly potent bifunctional peptide inhibitors in the suppression of experimental autoimmune encephalomyelitis. Clin Exp Immunol.

[26] Badawi AH, Kiptoo P, Wang WT, Choi IY, Lee P (2012). Suppression of EAE and prevention of blood-brain barrier breakdown after vaccination with novel bifunctional peptide inhibitor. Neuropharmacology.

[27] Ridwan R, Kiptoo P, Kobayashi N, Weir S, Hughes M (2010). Antigen-specific suppression of experimental autoimmune encephalomyelitis by a novel bifunctional peptide inhibitor: structure optimization and pharmacokinetics. J Pharmacol Exp Ther.

[28] Kobayashi N, Kiptoo P, Kobayashi H, Ridwan R, Brocke S (2008). Prophylactic and therapeutic suppression of experimental autoimmune encephalomyelitis by a novel bifunctional peptide inhibitor. Clin Immunol.

[29] Kobayashi N, Kobayashi H, Gu L, Malefyt T, Siahaan TJ (2007). Antigen-specific suppression of experimental autoimmune encephalomyelitis by a novel bifunctional peptide inhibitor. J Pharmacol Exp Ther.

[30] Youssef S, Stuve O, Patarroyo JC, Ruiz PJ, Radosevich JL (2002). The HMG-CoA reductase inhibitor, atorvastatin, promotes a Th2 bias and reverses paralysis in central nervous system autoimmune disease. Nature.

[31] Zhao H, Kiptoo P, Williams TD, Siahaan TJ, Topp EM (2010). Immune response to controlled release of immunomodulating peptides in a murine experimental autoimmune encephalomyelitis (EAE) model. J Controlled Rel.

[32] Malek TR (2003). The main function of IL-2 is to promote the development of T regulatory cells. J Leuko Biol.

[33] Sakaguchi S, Sakaguchi N, Asano M, Itoh M, Toda M (1995). Immunologic self-tolerance maintained by activated T cells expressing IL-2 receptor alpha-chains (CD25). Breakdown of a single mechanism of self-tolerance causes various autoimmune diseases. J Immunol.

[34] Thornton AM, Donovan EE, Piccirillo CA, Shevach EM Cutting edge: IL-2 is critically required for the in vitro activation of CD4+CD25+ T cell suppressor function. J Immunol.

[35] Maloy KJ, Powrie F (2005). Fueling regulation: IL-2 keeps CD4+ Treg cells fit. Nat Immunol.

[36] Piehl F (2014). A changing treatment landscape for multiple sclerosis: challenges and opportunities. J Internal Med.

[37] Wraith DC (2009). Therapeutic peptide vaccines for treatment of autoimmune diseases. Immunol Lett.

[38] van der Merwe PA, Bodian DL, Daenke S, Linsley P, Davis SJ (1997). CD80 (B7-1) binds both CD28 and CTLA-4 with a low affinity and very fast kinetics. The J Exp Med.

[39] Linsley PS, Greene JL, Brady W, Bajorath J, Ledbetter JA (1994). Human B7-1 (CD80) and B7-2 (CD86) bind with similar avidities but distinct kinetics to CD28 and CTLA-4 receptors. Immunity.

[40] Greene JL, Leytze GM, Emswiler J, Peach R, Bajorath J (1996). Covalent dimerization of CD28/CTLA-4 and oligomerization of CD80/CD86 regulate T cell costimulatory interactions. J Biol Chem.

[41] van der Merwe PA (2002). Formation and function of the immunological synapse. Curr Opi Immunol.

[42] Lee KH, Holdorf AD, Dustin ML, Chan AC, Allen PM (2002). T cell receptor signaling precedes immunological synapse formation. Science.

[43] Murray JS, Oney S, Page JE, Kratochvil-Stava A, Hu Y (2007). Suppression of type 1 diabetes in NOD mice by bifunctional peptide inhibitor: modulation of the immunological synapse formation. Chem Biol Drug Des.

[44] Cao W, Yang Y, Wang Z, Liu A, Fang L (2011). Leukemia inhibitory factor inhibits T helper 17 cell differentiation and confers treatment effects of neural progenitor cell therapy in autoimmune disease. Immunity.

[45] Zepp J, Wu L, Li X (2011). IL-17 receptor signaling and T helper 17-mediated autoimmune demyelinating disease. Trends Immunol.

[46] Serada S, Fujimoto M, Mihara M, Koike N, Ohsugi Y (2008). IL-6 blockade inhibits the induction of myelin antigen-specific Th17 cells and Th1 cells in experimental autoimmune encephalomyelitis. Proc Natl Acad Sci.

[47] Gabrysova L, Wraith DC (2010). Antigenic strength controls the generation of antigen-specific IL-10-secreting T regulatory cells. Eur J Immunol.

[48] Mazza G, Sabatos-Peyton CA, Protheroe RE, Herman A, Campbell JD (2010). Isolation and characterization of human interleukin-10-secreting T cells from peripheral blood. Human Immunol.

[49] Fu S, Zhang N, Yopp AC, Chen D, Mao M (2004). TGF-beta induces Foxp3 + T-regulatory cells from CD4 + CD25 - precursors. Amer J Transplant.

[50] Lassmann H, Raine CS, Antel J, Prineas JW (1998). Immunopathology of multiple sclerosis: report on an international meeting held at the Institute of Neurology of the University of Vienna. J Neuroimmunol.

[51] Peterson JW, Bo L, Mork S, Chang A, Trapp BD (2001). Transected neurites, apoptotic neurons, and reduced inflammation in cortical multiple sclerosis lesions. Annals Neurol.

[52] Baker D, Gerritsen W, Rundle J, Amor S (2011). Critical appraisal of animal models of multiple sclerosis. Multiple Sclerosis.

